# Effects of Dietary Inorganic Nitrate Supplementation on Exercise Performance in Patients With Heart Failure: Protocol for a Randomized, Placebo-Controlled, Cross-Over Trial

**DOI:** 10.2196/resprot.8865

**Published:** 2018-04-06

**Authors:** Mary N Woessner, Itamar Levinger, Christopher Neil, Cassandra Smith, Jason D Allen

**Affiliations:** ^1^ Institute of Health and Sport Victoria University Melbourne Australia; ^2^ Western Center for Health and Research Education Victoria University St Albans Australia; ^3^ Australian Institute for Musculoskeletal Science Department of Medicine-Western Health Melbourne Medical School, The University of Melbourne Melbourne Australia; ^4^ Department of Medicine-Western Health University of Melbourne Melbourne Australia; ^5^ Department of Kinesiology University of Virginia Charlottesville, VA United States

**Keywords:** cardiovascular disease, nitric oxide, exercise tolerance

## Abstract

**Background:**

Chronic heart failure is characterized by an inability of the heart to pump enough blood to meet the demands of the body, resulting in the hallmark symptom of exercise intolerance. Chronic underperfusion of the peripheral tissues and impaired nitric oxide bioavailability have been implicated as contributors to the decrease in exercise capacity in these patients. nitric oxide bioavailability has been identified as an important mediator of exercise tolerance in healthy individuals, but there are limited studies examining the effects in patients with chronic heart failure.

**Objective:**

The proposed trial is designed to determine the effects of chronic inorganic nitrate supplementation on exercise tolerance in both patients with heart failure preserved ejection fraction (HFpEF) and heart failure reduced ejection fraction (HFrEF) and to determine whether there are any differential responses between the 2 cohorts. A secondary objective is to provide mechanistic insights into the 2 heart failure groups’ exercise responses to the nitrate supplementation.

**Methods:**

Patients with chronic heart failure (15=HFpEF and 15=HFrEF) aged 40 to 85 years will be recruited. Following an initial screen cardiopulmonary exercise test, participants will be randomly allocated in a double-blind fashion to consume either a nitrate-rich beetroot juice (16 mmol nitrate/day) or a nitrate-depleted placebo (for 5 days). Participants will continue daily dosing until the completion of the 4 testing visits (maximal cardiopulmonary exercise test, submaximal exercise test with echocardiography, vascular function assessment, and vastus lateralis muscle biopsy). There will then be a 2-week washout period after which the participants will cross over to the other treatment and complete the same 4 testing visits.

**Results:**

This study is funded by National Heart Foundation of Australia and Victoria University. Enrolment has commenced and the data collection is expected to be completed in mid 2018. The initial results are expected to be submitted for publication by the end of 2018.

**Conclusions:**

If inorganic nitrate supplementation can improve exercise tolerance in patients with chronic heart failure, it has the potential to aid in further refining the treatment of patients in this population.

**Trial Registration:**

Australian New Zealand Clinical Trials Registry ACTRN12615000906550; https://www.anzctr.org.au/Trial/Registration/TrialReview.aspx?id=368912 (Archived by WebCite at http://www.webcitation.org/6xymLMiFK)

## Introduction

### Background

Chronic heart failure (CHF) is a condition characterized by the inability of the heart to pump sufficient blood to meet the metabolic demands of the body. Affecting over 23 million people worldwide, this disease is the leading cause for hospital admission in both Europe and the United States [1]. CHF is a multifarious syndrome that presents with different physiological impairments depending on age, medical history, pathology, and left ventricular ejection fraction status [2]. Although the etiology of CHF may vary, patients are all plagued by the hallmark symptoms of exercise intolerance (low aerobic capacity), dyspnea, and fatigue [3].

Exercise intolerance, defined by a reduced peak oxygen uptake (VO_2peak_), independently predicts morbidity and mortality and directly contributes to a reduced quality of life in patients with CHF [4-6]. In comparison with healthy controls, patients with CHF have significantly lower VO_2peak_ (~13.5ml/kg/min vs ~23.8ml/kg/min), with accompanying reductions in cardiac output (CO) by 52-53% during maximal exercise [7-9]. Although it was historically assumed that this inability to augment CO during exercise was the primary contributor to exercise intolerance, more recent investigations suggest that resulting under-perfusion of the peripheral muscular tissues may have a more detrimental impact [3,10].

Following acute heart failure, there is an increased activation of the sympathetic nervous system (SNS), which leads to vasoconstriction of arteries supplying blood to the peripheral tissues to defend central blood pressure and vital organ perfusion [10]. Although this SNS response is critical initially, continued overactivation results in chronic underperfusion of the skeletal muscle tissues, thereby contributing to capillary density rarefaction and a preferential loss of type-I oxidative fibers, and thus shifting these patients to a more glycolytic phenotype [11-14]. In the heart and skeletal muscles, there are significant abnormalities in the mitochondrial function, leading to decreases in oxidative phosphorylation [15]. Within the vasculature, a reduction in nitric oxide (NO) bioavailability is also highly prevalent in CHF and has been correlated with both the severity of CHF and the patients’ functional capacity [16]. NO is a key regulator of blood flow and as large and small vessel vasodilation is a crucial contributor to exercise capacity, the inability of patients with CHF to up-regulate NO could be a limiting factor in their exercise tolerance [3,17]. This knowledge has brought about a fundamental shift in the treatment focus for CHF, whereby interventions are now targeting improvements within the peripheral tissue function to restore exercise tolerance.

One emerging therapeutic approach is supplementing with dietary inorganic nitrate (found in kale, green leafy vegetables, or beetroot juice [BTR]) to increase circulating NO bioavailability [18]. This occurs via a 2-step process, whereby nitrate is swallowed and absorbed via the gut and released into circulation. Approximately 25% of nitrate becomes highly concentrated in the salivary glands, which is then secreted and subsequently reduced via oral commensal bacteria to nitrite, which is then swallowed and absorbed into the circulation [19]. The circulating nitrite in the plasma may act as a relatively protected NO species that can be reduced to NO in low-oxygen environments (such as in tissues with low partial pressure of oxygen or during exercise).

Studies in healthy populations have demonstrated a myriad of benefits in exercise performance following nitrate supplementation, including increases in time to exhaustion, oxygen consumption efficiency (during submaximal exercise), total power output, and decreased systemic blood pressure [20-27]. Inorganic nitrate supplementation has even greater potential efficacy in clinical populations as supplementation may be an effective way of assisting in the targeted redistribution of blood flow in the underperfused peripheral tissues [18]. However, in contrast to the numerous studies in healthy populations, there are relatively few studies to date that have examined the effects of inorganic nitrate supplementation on exercise capacity in clinical patients, and only 4 have been on the CHF population [28-31]. A further limitation in our current understanding of inorganic nitrate supplementation in patients with CHF is the lack of substantial evidence in each of the 2 individual classifications of CHF, with a large proportion of the publications focusing only on one classification.

Currently, there are 2 classifications for patients with heart failure differentiated by whether the patient has a preserved ejection fraction (HFpEF, also known as diastolic dysfunction) or a reduced ejection fraction (HFrEF, also known as systolic dysfunction). HFrEF often results from an acute ischemic event that causes tissue death, leaving the cardiac muscle less able to contract adequately. In these patients, the ejection fraction is reduced because of left ventricular chamber dilation [32,33]. Patients with HFrEF have a lower CO both at rest and during exercise as compared with HFpEF patients and healthy controls [8]. HFpEF typically has a slower onset and these patients are more likely to be older, female, and suffer from a myriad of other comorbidities. Although they also have significant left ventricular remodeling, in HFpEF, the chamber size remains unchanged, but there are increases in the wall thickness and the ratio of ventricular mass to chamber volume. These maladaptations lead to significant elevations in LV filling pressures, which is known to cause exertional dyspnea and further contribute to exercise intolerance [34]. Additionally, the impaired arterial hemodynamic profile of these patients (increased arterial stiffness, reduced exercise induced vasodilation) creates a unique model for which a vasodilatory intervention, such as inorganic nitrate supplementation, could be very effective [35,36].

In CHF, the HFpEF cohort has been the most studied. Zamani et al provided acute supplementation of inorganic nitrate in the form of beetroot juice (12.9 mmol nitrate) to 17 HFpEF patients and saw improvements in total time to exhaustion (TTE) and VO_2peak_ during a maximal exercise test [29]. The performance benefits were accompanied by an increase in CO, although this was secondary to a decrease in systemic vascular resistance [29]. Following chronic inorganic nitrate dosing, Eggebeen et al found that 6.1 mmol nitrate/day for 7 days (in the form of beetroot juice) led to a 24% increase in TTE during an exercise bout of cycling at 75% of each individual’s maximum power output [28]. Similarly, Zamani et al identified significant increases in TTE following a 2-week potassium nitrate dosing regimen (6 mmol/day for 1 week, increasing to 18 mmol/day for the second week) [37]. These studies lend support to inorganic nitrate supplementation’s potential efficacy for improving exercise tolerance in patients with HFpEF; however, the small sample sizes and limited mechanistic data leave plenty of scope for future studies.

In comparison with the positive results seen in HFpEF cohorts, data for HFrEF remains limited. A recent study by Hirai et al supplemented HFrEF patients with 12.9 mmol nitrate/day in the form of beetroot juice for 9 days and reported no changes in any of the parameters examined, including exercise performance, central hemodynamics, and blood pressure [31]. The authors suggest that the negative findings could be because HFrEF patients have relatively normal oxygen extraction rates within the peripheral tissues. Although Hirai et al is the only human study examining the effects of inorganic nitrate supplementation on exercise capacity in patients with HFrEF, Coggan et al conducted a study examining the effects of nitrate supplementation on isokinetic knee extensor power [30]. This study showed a 13% increase in the maximal power output following a single dose of inorganic nitrate (11.2 mmol). The authors suggested that the substantial improvement (they note that it is much larger than the 6% increase observed in healthy controls) was due to NO’s known effect of increasing the activation of cyclic guanosine monophosphate. As this activation is known to lead to increases in maximal power output, particularly in type II fibers, it lends further support to nitrate’s efficacy in CHF (where patients are known to be more type II-fiber dominant). In further support of these findings, 2 separate CHF rat model studies, 1 using an acute dose (5 mg/kg sodium nitrite) and 1 using a chronic dose (1 mmol nitrate/kg/day for 5 days), demonstrated significant increases in blood flow and vascular conductance in skeletal muscle [38,39]. The acute sodium nitrite infusion also showed a preferential increase in blood flow, specifically in the muscles of the rats with a higher percentage of both type IIb + IId/x fast-twitch fibers [38]. This further illustrates the potential efficacy of nitrate supplementation within the HFrEF population. Thus, despite the lack of positive findings in the study by Hirai et al, there remains a lot of promise in the use of nitrate supplementation in patients with HFrEF.

Overall, the area of inorganic nitrate supplementation in patients with CHF is relatively new. The current studies have small sample sizes (3 of the 5 human studies had ≤12 participants) that lack diversity in gender (Hirai et al only had male participants) and testing modalities (all exercise studies used cycle ergometry). Moreover, no study to date has sought to examine and compare the effects of nitrate supplementation in HFrEF and HFpEF patients within the same testing protocol. The development of research trials recruiting both HFrEF and HFpEF patients is critical to advancing our understanding of how to best clinically target and treat the mechanistic differences between these 2 distinct classes of CHF.

### Objective

The primary aim of this study is to test the hypothesis that 5 days of inorganic nitrate supplementation (16 mmol/day) will improve exercise tolerance (VO_2peak_ and TTE) in both HFpEF and HFrEF patients. We further hypothesize that patients with HFpEF will have larger improvements in exercise tolerance than patients with HFrEF because of greater impairments in their peripheral muscular tissues. The secondary aim of the project is to identify the mechanistic contributors to exercise tolerance in both heart failure classifications via examining the following outcome measures:

1. Gastrocnemius tissue oxygenation at rest and during submaximal and maximal exercise via near-infrared spectroscopy (NIRS)
2. Vastus lateralis muscle tissue composition and function (angiogenesis, capillaries per unit area and per muscle fiber, mitochondrial function, and muscle fiber composition)
3. Vascular function via brachial artery flow-mediated dilation, lower-limb blood flow via plethysmography, and pulse wave velocity (PWV) and reflection.

## Methods

### Study Design and Participants

**Figure 1:**
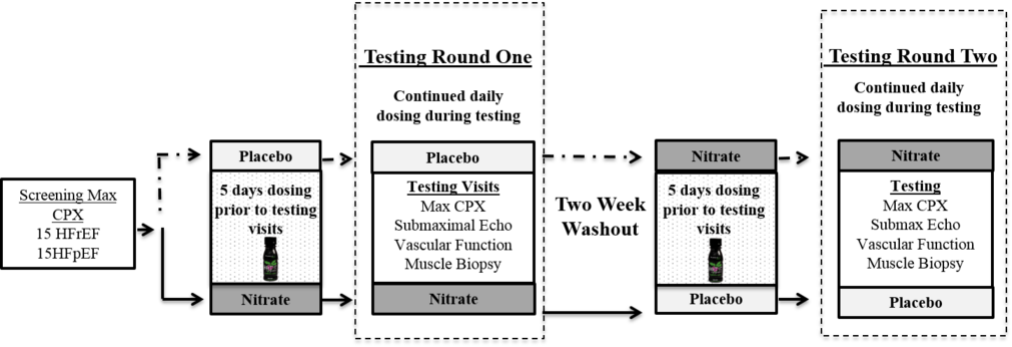
Max CPX: maximal cardiopulmonary exercise test; submaximal echo: submaximal exercise test with echocardiograph imaging; vascular testing: resting measures of blood flow and arterial function; and muscle biopsy: vastus lateralis muscle biopsy.

This is a randomized, double-blind, placebo-controlled, crossover study (see Figure 1). Following a screening visit, participants will be randomized to consume either nitrate-rich beetroot juice or a nitrate-depleted placebo for 5 days. Following this 5-day loading, the participants will continue daily dosing until the completion of the 4 testing visits (maximal cardiopulmonary exercise test (CPX), submaximal exercise test with echocardiography, vascular function assessment, and vastus lateralis muscle biopsy). Due to the need for adequate rest days between exercise visits, it could take up to 2 weeks in total to complete all testing visits within each round. However, both the dosing days and testing order will be matched within each participant between the 2 rounds of supplementation. Participants will then have a 2-week washout period before completing the second round of the study.

### Recruitment Strategies and Eligibility

We aim to recruit 15 patients with HFrEF and 15 patients with HFpEF. A total sample of 30 is a realistic number of patients that can be recruited within the time frame of the study (18 months) and is similar to other successful studies in this field [29,40].

Recruitment will be open to individuals between the ages of 40 and 85 years who have diagnosed stable CHF with either HFrEF ≤40% or HFpEF ≥50% with no major changes in medications for at least 3 months (see Textboxes 1 and 2).

**Inclusion criteria.**

1. Aged between 40 and 85 years
2. Diagnosed stable chronic heart failure (CHF) with either reduced ejection fraction (HFrEF) ≤40% or preserved ejection fraction (HFpEF) ≥50%
3. New York Heart Association class II-III
4. On stable medications for at least 3 months
5. Peak VO_2_ <85% of age-predicted max

Additional criteria for HFpEF recruitment

1. Evidence of abnormal diastolic filling pressure (eg, abnormal E/e′, abnormal deceleration time, dilated left atrial volume, or elevated brain natriuretic peptide [if available]) [41]
2. Signs and symptoms of heart failure, plus definite episodes of decompensated heart failure (adjudicated via Boston Criteria)

**Exclusion criteria.**

1. A major cardiovascular event within the previous 6 weeks or a planned hospitalization within the next 2 months
2. Patients with an ejection fraction between 41 and 49
3. Uncontrolled diabetes (>9% HbA1C [glycated hemoglobin])—Can delay start of testing by 3 months until levels are controlled and stable
4. Foot ulcers/advanced neuropathy or other musculoskeletal condition that could limit exercise performance
5. Abnormal response to CPX
6. Allergy to beets or proton pump inhibitors
7. Refusal or inability to abstain from the use of proton pump inhibitors for 24 hours before testing

Potential participants will be identified through medical chart reviews and will be contacted in person during a hospital or clinic visit. They will be given the contact information for the trial recruitment coordinator and instructed to contact the team if they wish to learn more about the study. At all stages, potential participants will be reminded that their participation in the study is voluntary and that their decision to participate or not will in no way affect their usual care.

### Screening Visit

Patients who wish to participate in the study will be asked to sign an informed consent. They will then complete a screening maximal CPX that will be supervised by a medical practitioner. Although the primary purpose of CPX is to screen for adverse events or contraindications to participation in the study, it will also serve as a familiarization visit for the participants. This CPX employs a 2-step protocol that includes 6 min of low-intensity walking at 1.4 km/hour at a 4% grade. Following this, the speed and/or incline will be increased in an individualized manner as the participants’ capabilities allow until maximal exertion is achieved. The test will only be stopped if the medical practitioner deems it unsafe to continue or the patient requests to stop. The max CPX protocol will be kept constant between visits for each individual subject. Similar protocols have been previously used in this population as the 2-step protocol allows for collection of both submaximal and maximal measures of aerobic capacity and function within 1 exercise bout [42-44].

### Supplementation

Following successful completion of the screening CPX, participants who meet the inclusion criteria will be randomly allocated in a double-blind fashion to determine the order of treatment (beetroot juice or placebo). A technical staff member of Victoria University, external to the project, will color code the beetroot juice bottles and provide a randomization sheet to the research team that has the conditions removed and replaced with colors.

Participants will consume three 70-ml bottles of beetroot juice (BEET IT shot, James White Drinks, Ipswich, UK) per day of either a nitrate-rich beetroot juice (16 mmol of nitrate/day total) or a nitrate-depleted placebo for 5 days before they commence testing. They will then continuously dose until they complete all 4 testing visits. There will be a 2-week washout period between the 2 rounds. As the half-life of nitrate is 5-8 hours, a 2-week washout period should be sufficient to minimize any possible residual effect of nitrate [45]. The order of testing visits will be kept consistent for each participant between the beetroot juice and placebo testing rounds. On testing days, patients will consume the first bottle of either beetroot juice or placebo 2.5 to 3 hours prior, as plasma nitrite concentration peaks within 2.5 to 3 hours postingestion [23,24]. To assess supplementation compliance, participants will maintain a dosing log and return all bottle caps to the research team.

The cumulative body of literature identifies that decreases in the oxygen cost of exercise and increases in power output and time to exhaustion were seen in studies using a minimum dose of 5.2 mmol nitrate for 6 days [22,25-27]. A longer dosing protocol (15 days) demonstrated that improvements in steady-state VO_2_ seen at day 5 were maintained but not increased at day 15 [46]. A previous clinical trial has demonstrated that an 18.1 mmol nitrate/day dose was feasible and safe for patients with peripheral arterial disease, whereas doses as high as 12.9 mmol nitrate/day have been used in the CHF population [40,47,48]. Thus, the dosing amount (16 mmol nitrate) and duration (minimum of 5 days before first testing session) for this study were selected to maximize the potential effects of the nitrate supplementation.

### Quality of Life and Health Status Questionnaires

In addition to the physiological measures attained, participants will be asked to complete a series of questionnaires through the study, including the Minnesota Living with Heart Failure Questionnaire (MLHFQ) to determine how their CHF has affected their life during the last month; the Subjective Exercise Experience Scale (SEES) to assess the effect of an acute exercise bout on positive well-being, psychological distress, and fatigue; and finally, they will complete a Physical Activity Questionnaire (PAQ) to confirm their current level of physical activity [49,50].

Participants will be asked to complete MLHFQ and PAQ a total of 2 times, once before commencing each testing round to ensure there is no change in how their CHF affects their daily life and how much they are exercising. MLHFQ has been previously validated for the use of the resulting physical, emotional, and total scores in patients with CHF [50]. Participants will be asked to complete SEES after every exercise test for the duration of the study, including the screening CPX. This 12-question scale was developed and validated as a 3-factor measure (positive well-being, psychological distress, and fatigue) of psychological response to exercise and has been previously validated [51].

### Maximal Cardiopulmonary Exercise Test Testing Visit

Participants will complete a 2-phase treadmill test identical to the screening CPX but with the addition of blood draws at rest and 10 min into recovery (to allow for postexercise blood volume stabilization) to quantify both resting plasma nitrate/nitrite as well as postexercise nitrate/nitrite changes.

Upon arrival for the testing visit, a catheter will be inserted into the antecubital vein of the participant to allow for repeated blood sampling. At this visit, 30 ml of blood will be collected in total. Following the resting blood draw, participants will be fitted with a 12-lead electrocardiograph to monitor their heart rhythms throughout the maximal test. Additionally, a near-infrared spectrometry (NIRS, PortaMon, Artinis Medical Systems B.V., The Netherlands) device will be placed on the skin above the gastrocnemius muscle of the participant. The NIRS system is noninvasive and provides an assessment of tissue oxygenation via the transmission of specific wavelengths of light (850 nm and 764 nm) that are absorbed by oxy- and deoxyhemoglobin, respectively [52]. A detection probe within the device measures the intensity of the received and transmitted light, which is communicated to a laptop via Bluetooth, and the corresponding software calculates the relative concentrations (and relative change) of oxygenated and deoxygenated hemoglobin within the muscle tissue. The device will be placed on the widest part of the medial head of the gastrocnemius, which is located by having the participant perform a short series of calf raises. A measuring tape will be used to identify the vertical point on the calf corresponding to the widest girth. A skinfold measurement will be taken at the selected site to ensure that the adipose tissue is less than 1.5 cm (typically the calf has less than 1 cm) [53]. The site will then be prepped with alcohol wipes (and shaving if appropriate), and the device will be affixed with micropore tape. Once the device is placed, vertical measurements are then taken from the top of the device to the bottom of the medial malleolus for reproducibility purposes on future tests. To ensure no light enters the NIRS device, a black plastic will be wrapped over the device and taped into place.

Additionally, a PhysioFlow (Physio Flow; Manatec Biomedical; Macheren, France) device will be used to estimate CO and the systemic vascular resistance index both at rest and during exercise. The device is a noninvasive hemodynamic monitor that provides real-time calculations of CO and various other parameters based on the morphological analysis of the bioelectrical impedance waveform. This device has been previously validated for measures of CO in healthy populations, but the studies in clinical populations thus far have been limited [54-57].

After 10 min of resting data collection, participants will be asked to complete a 2-step maximal treadmill CPX (as in the screening visit). Upon completion, the participant will be seated for recovery and at 10-min postcompletion will have a final 5 ml of blood drawn. Additionally, to allow for comparisons of the NIRS results to be made between subjects, a postexercise physiological calibration will be used to convert the relative concentration values to a normalized scale. For this, an occlusion cuff will be applied to the NIRS leg just above the knee, and the patients will undergo 5 min of ischemia. The baseline value for the scale will be the plateau of the oxygenated hemoglobin signal, whereas the signal response to postcuff release will provide a functional maximum for the normalized scale. All values obtained during the testing will then be expressed as a percentage value within these ranges [58].

### Vascular Function Visit

Patients will be asked to fast overnight and to abstain from exercise for the 24 hours prior and to avoid caffeine and smoking for the 3 hours before testing. They will also be asked to hold their morning medications until immediately post-testing. All vascular testing will occur following a minimum of 10 min, with the participant in the supine position.

Brachial artery flow-mediated dilation (FMD) will be obtained using a high-resolution Terason ultrasound (LifeHealthcare, New South Whales, Australia) to capture images of the brachial artery. This method has been previously used in clinical populations and has been shown to be reliable [40,59,60]. In brief, FMD will be assessed at baseline, following 5 min of forearm occlusion, and 2 min following occlusion cuff release (reactive hyperemia). These data points will be used to calculate the percentage of change in brachial artery diameter following reactive hyperemia.

Lower limb blood flow will be assessed via venous occlusion strain gauge plethysmography both at rest and during reactive hyperemia following 5 min of occlusion via Hokanson A16 (DE Hokanson, Bellevue, WA), as was previously described [61,62]. Participants will remain in a supine position with their legs elevated (to facilitate venous emptying) for the duration of the test. A cuff will be placed on the upper thigh of the nonbiopsy leg (so as not to put pressure on the biopsy site) to act both as a venous and arterial occlusion cuff while a mercury strain gauge (sized at ~4 cm less than calf width) is affixed around the largest part of the gastrocnemius. For resting measures, the thigh cuff will be inflated to 50 mmHg for 4 to 6 cycles of inflation and deflation to obtain resting blood flow measures. Resting blood flow will be recorded as the average of 3 measurements. Peak hyperemic blood flow will be determined following an ischemic occlusion (pressure set to 30 mmHg above systolic pressure) of the thigh for a period of 5 min. Postocclusion blood flow measurements were obtained every few seconds following cuff release, with the peak value being recorded as the highest value achieved.

Vascular stiffness will be assessed by PWV and pulse wave reflection using applanation tonometry via a SphygmoCor XCEL system (AtCor Medical, New South Whales, Australia. The SphygmoCor is a noninvasive diagnostic system that has been used to provide assessments of both the central blood pressure and PWV of clinical patients [63]. For the measurement of pulse wave analysis (PWA), a SphygmoCor arm cuff is placed on the upper arm, aligning the designated markings with the brachial artery. The system then measures pulsations recorded at the brachial artery to produce central aortic pressure waveforms and predict the following: central systolic pressure, central pulse pressure, augmentation pressure, and augmentation index. PWV is measured via a simultaneous comparison of the carotid and femoral arterial pulses. A thigh cuff will be placed around the participant’s upper thigh, which acts to measure the femoral pulse via pulsations, while simultaneously a tonometer will be used to assess the carotid pulse. Higher pulse wave velocities from the carotid to femoral arteries indicate higher aortic stiffness.

### Submaximal Echocardiograph Visit

For this visit, participants will again be asked to refrain from exercise and alcohol for the 24 hours before testing and to abstain from caffeine and smoking for the 3 hours before testing. They will be instructed to follow their normal dietary routine and take their medications. For this visit, participants will complete a series of 3 discontinuous stages (5-min rest in between each stage) of exercise on an echo-compatible recumbent cycle ergometer (Vivid 7 echocardiographic machine, GE, Milwaukee, Wisconsin). At present, the most common measure to assess the heart function during exercise is to have the participant exercise and then capture images immediately after exercise completion. The design of this cycle places the participant in an ideal position to capture echocardiograph images during exercise, allowing for more accurate assessment of cardiac function during exercise. Three independent workloads will be chosen based on participant capacity (Stage 1: 5-20 watts, Stage 2: 15-40 watts, and Stage 3: 30-60 watts). During the exercise test, participants will be fitted with the NIRS device, PhysioFlow, and electrocardiogram, similar to the one used during the max CPX. The echocardiograph will provide measures of CO, stroke volume (SV), mean arterial pressure (MAP), cardiac power output, left-ventricular end-systolic elastance, arterial elastance, preload recruitable stroke work, long-axis contraction and relaxation, mitral flow propagation velocity, and tricuspid incompetence. All echocardiograph assessments will be taken by the same tester to control for intertester variability.

### Vastus Lateralis Muscle Biopsy Visit

The biopsy will be performed in a similar fashion to previous studies by our group on a separate day with at least 48 hours recovery between other testing visits [64,65]. In brief, the participant will be placed in a supine position. Following an injection of local anesthetic into the skin and fascia (1% Xylocaine), a small incision will be made at the level of the left vastus lateralis. A muscle sample will be taken (~150-300 mg wet weight) using a Bergström biopsy needle with manual suction applied [66]. Once obtained, muscle samples will be processed; cleaned of excess blood, fat, and connective tissue; and split into 3 portions. One portion (10-20 mg) will be immediately immersed in a 5-ml tube containing ~3 ml of biopsy-preserving solution kept on ice and used for in situ measurements of mitochondrial respiration. The second portion (around 20 mg) will be imbedded with Tissue-Tek for the immunohistochemistry analysis. The samples will be immediately frozen in liquid nitrogen and stored at −80°C for subsequent analyses.

### Ethical Considerations

This study has been approved by the Melbourne Health [HREC/15/MH/166] and Victoria University Ethics Committees. The trial has been registered in the Australian New Zealand Clinical Trials Registry [ACTRN12615000906550].

### Outcome Measurements

The primary outcome measure will be exercise tolerance [VO_2peak_ and TTE] during the 2-step CPX test. Secondary outcomes will include measures at rest and during exercise for cardiac function (CO, SV, MAP), as well as peripheral tissue function (lower limb blood flow, endothelial function, PWA, PWV, mitochondrial function). In addition, measures of plasma nitrate and nitrite will be recorded for nitrate-nitrite conversion rate calculations.

### Complications and Adverse Events

Although complications and adverse events associated with the intervention are unlikely, participants will be asked to self-report any symptoms or adverse events they experience. The only previously documented side effects are beeturia (red urine) and red stools [23,67]. Any adverse events noted by the researchers or participants will be reported in the final manuscript.

### Statistical Analysis

The primary endpoint of this pilot study is exercise capacity (VO_2peak_ and TTE) during the maximal CPX in both the HFpEF and HFrEF cohorts. A repeated-measures *t* test will be conducted to determine the changes in exercise capacity variables in the 2 groups combined. This will be followed by a repeated measure analysis of variance to determine the time×group effect in HFpEF and HFrEF patients. Similar analyses will be performed on the secondary endpoints/variables listed in the Specific Aims. Post hoc comparisons of change scores for relevant variables between HFpEF and HFrEF will be performed. Additional pairwise tests as well as linear regressions between the placebo and beetroot juice conditions will be used to determine what physiological factors (mitochondrial efficiency, endothelial function, tissue perfusion, leg blood flow, CO) contribute to any potential changes in exercise tolerance in HFpEF versus HFrEF.

## Results

Data collection from this paper is currently underway. Predicted completion of the recruitment phase is mid-2018.

## Discussion

### Principal Findings

Heart failure is a chronic, progressive condition that has deleterious effects on both the central and peripheral function of the body, resulting in the hallmark symptom of exercise intolerance. This decrease in aerobic capacity is linked with lower rates of survival, a higher burden of disability, and increased rates of hospitalization for patients with CHF and is a prime target for rehabilitative interventions [4,68]. Thus, interventions that can acutely improve the tolerability of exercise for these patients could represent a crucial step forward for treatment.

The proposed project will be the first to comprehensively compare the central and peripheral function at rest and during exercise in both HFpEF and HFrEF patients on and off nitrate supplementation. This study will also be the first study in CHF that assesses the effects of nitrate supplementation within the skeletal muscle tissue (mitochondrial function, capillary density, and muscle fiber composition). Results from previous trials (in healthy individuals) have indicated the potential beneficial impact of nitrate supplementation on mitochondrial function, but this mechanistic change has yet to be demonstrated in CHF patients [21].

### Limitations

The sample size for this study, although larger than some comparable trials, is still quite small. However, the crossover design helps to reduce the potential variability and improve the possibility of detecting differences between both the placebo and beetroot juice groups and the HFpEF and HFrEF groups. The high chronic dose was chosen to maximize the potential benefits of the supplementation, but the authors note that the amount is much higher than what would be consumed naturally in the diet. Finally, although every effort will be made to keep the supplementation days consistent before each testing visit between the placebo and beetroot juice rounds, because of patient and medical team availability, it is not possible to fully control this aspect. The 5-day loading period before any testing should help to standardize the dosing days. However, we will report any difference in dosing days between the 2 treatment arms.

### Conclusions

Our understanding of the potential effects of nitrate supplementation in patients with CHF is still in its infancy. Although the initial studies show promise, the studies have been small and limited in scope to just 1 classification of CHF. This is the first study to directly compare the effects of inorganic nitrate supplementation on patients with HFrEF and HFpEF following an identical protocol to tease out any changes (and/or differences in changes) in peripheral and central factors. This is a critical step in advancing our current knowledge of CHF as a disease as well as the efficacy of nitrate supplementation. Given the relationship between exercise capacity and mortality and morbidity, if inorganic nitrate supplementation can improve exercise tolerance in patients with CHF, it has the potential to aid in further refining the treatment of patients in this population.

## Abbreviations

CHF: chronic heart failure
CO: cardiac output
CPX: cardiopulmonary exercise test
FMD: flow-mediated dilation
HFpEF: heart failure preserved ejection fraction
HFrEF: heart failure reduced ejection fraction
MAP: mean arterial pressure
MLHFQ: Minnesota Living With Heart Failure Questionnaire
NIRS: near-infrared spectroscopy
NO: nitric oxide
PAQ: Physical Activity Questionnaire
PWA: pulse wave analysis
PWV: pulse wave velocity
SEES: Subjective Exercise Experience Scale
SNS: sympathetic nervous system
SV: stroke volume
TTE: time to exhaustion
VO2peak: peak oxygen consumption
